# 2350 Creating a comprehensive municipal inventory of common ragweed (*Ambrosia artemisiifolia*) to predict allergenic pollen exposures

**DOI:** 10.1017/cts.2018.56

**Published:** 2018-11-21

**Authors:** Daniel S. W. Katz, Stuart Batterman

**Affiliations:** University of Michigan School of Medicine

## Abstract

OBJECTIVES/SPECIFIC AIMS: One of the key difficulties in predicting allergenic pollen exposures has been a lack of information on source plant location and abundance. However, the increasing availability of spatially explicit data from remote sensing offers new opportunities to create comprehensive inventories of allergenic pollen producing plants. METHODS/STUDY POPULATION: In this study, we use a spatially oriented field survey to map common ragweed (*Ambrosia artemisiifolia*) in Detroit, MI, USA. We then combine this with remote sensing imagery and LiDAR to predict ragweed presence and potential pollen production across 344 km^2^ of Detroit. Finally, we compare this with measurements of airborne pollen concentrations collected throughout the city. RESULTS/ANTICIPATED RESULTS: Our initial results show that ragweed is present in ~2% of the city, and its presence and abundance are strongly associated with demolished building (*p*<0.001). The uneven distribution of ragweed plants across the city leads to substantially higher pollen concentrations in neighborhoods where more buildings have been recently demolished. DISCUSSION/SIGNIFICANCE OF IMPACT: Our approach offers an effective way to quantify allergenic pollen production, airborne concentrations, and exposures across a large metropolitan area. This in turn provides insight on how to best reduce airborne pollen concentrations: in this case, by changing post-demolition land management practices.

